# The application of radiomics in the diagnosis and evaluation of cognitive impairment related to neurological diseases

**DOI:** 10.3389/fnins.2025.1591605

**Published:** 2025-07-02

**Authors:** Haixing Xiao, Xinyi He, Wei Zhou, Xianghong Guo, Xiuying Cai, Tan Li

**Affiliations:** ^1^The First Affiliated Hospital of Soochow University, Suzhou, Jiangsu, China; ^2^Suzhou Industrial Park Xinghai Hospital, Suzhou, Jiangsu, China

**Keywords:** cognitive impairment, radiomics, Alzheimer’s disease, Parkinson’s disease, cerebral small vessel disease, stroke

## Abstract

Cognitive impairment (CI) is common, with diverse underlying causes, symptoms, and imaging features. It often leads to disability and loss of independence. Early diagnosis and assessment of CI are crucial for the prognosis improvement. Conventional diagnostic methods for CI are hindered by subjectivity and imprecision. Radiomics, a sophisticated and objective methodology, has been increasingly utilized in CI in recent times. This article describes the methodology of radiomics and reviews the application of radiomics in the prediction and evaluation of cognitive impairment related to neurological diseases such as Alzheimer’s disease (AD), Parkinson’s disease (PD), cerebral small vessel disease (CSVD), and stroke. It can provide imaging markers for the early diagnosis and risk stratification of cognitive impairment.

## 1 Background

As the global population ages, the incidence of cognitive impairment (CI) and dementia is increasing significantly. Studies indicate that approximately one in five individuals over the age of 65 will experience varying degrees of cognitive decline (Han et al., 2020). CI encompasses a complex symptomatology associated with various diseases and may lead to disability and compromised independent living capacity in affected individuals. Growing evidence suggests that dementia can be significantly mitigated through early diagnosis and intervention (Reuben et al., 2024). Traditional diagnostic methods for cognitive impairment (CI) primarily rely on clinical assessments and standardized scales, which are often constrained by subjectivity and limited precision. In this context, radiomics emerges as an advanced and complementary technique for the diagnosis and evaluation of CI.

## 2 Overview of radiomics

### 2.1 Introduction

Radiomics is an imaging technology, which extracts a large number of quantitative features based on the medical imaging data through computer science and statistical methods to reveal potential associations between images and diseases, and to support the diagnosis, prognosis assessment and treatment of diseases. It is firstly proposed by Lambin et al. (2012). This technology excels in identifying imaging features or biomarkers associated with specific diseases through systematic analysis of high-dimensional imaging data and textural features (Gillies et al., 2016). Radiomics analysis encompasses a diverse spectrum of analytical approaches, including voxel-based methods, connectivity-based techniques, and pattern recognition-based strategies. The radiomics analysis process typically involves several key steps: image data acquisition, region of interest (ROI) segmentation, feature extraction, feature selection, model training, and ultimately classification or prediction (Bera et al., 2022; Lambin et al., 2012), Figure 1 depicts a radiomics workflow diagram and provides additional methodological details.

**Figure 1:**
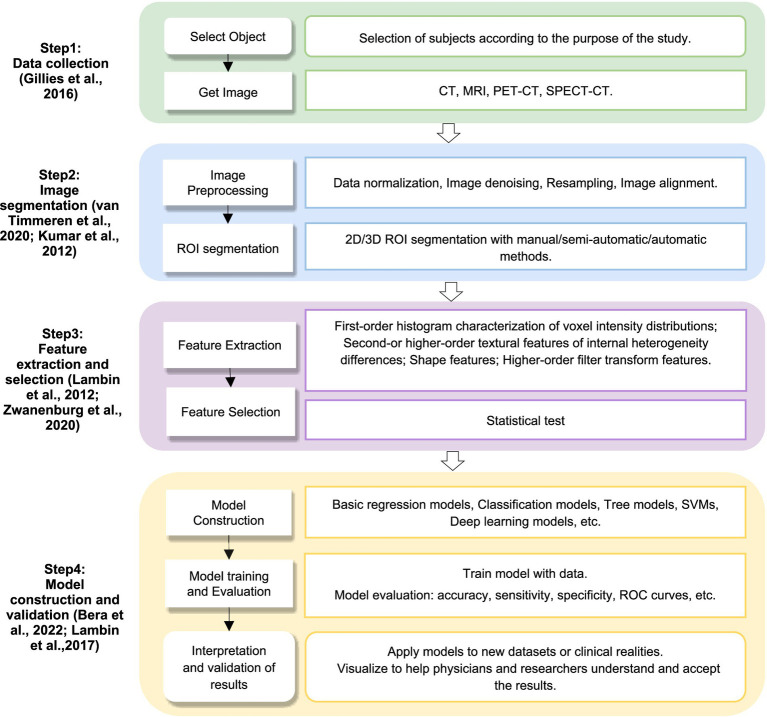
Radiomics workflow. CT, computed tomography; MRI, magnetic resonance imaging; PET-CT, positron emission tomography-computed tomography; SPECT–CT, single-photon emission computed tomography-computed tomography; ROI, region of interest; SVM, support vector machine; ROC, receiver operation characteristic.

### 2.2 Segmentation of region of interest

Segmentation of ROI facilitates targeted analysis of specific brain regions or structures. The methodologies for ROI segmentation can be categorized into several distinct approaches (Cardenas et al., 2019; van Timmeren et al., 2020): (1) Manual labeling: Researchers manually delineate the boundaries of the ROI by selecting and segmenting specific regions. This approach is well-suited for limited datasets and less complex anatomical structures. However, it exhibits limitations in both computational efficiency and anatomical precision when dealing with large datasets or complex brain regions (Kumar et al., 2012; Zwanenburg et al., 2020). (2) Standard templates: This method employs validated standard brain templates, such as those from the Montreal Neurological Institute (MNI) (Pan et al., 2022), whereby researchers spatially normalize these templates to individual brain images and segment the ROI based on predefined regions. While effective for normative brain regions, this method may not account for individual neuroanatomical variations, thereby potentially compromising segmentation accuracy in specific brain regions. (3) Automatic segmentation algorithms: These algorithms implement advanced image processing techniques to autonomously localize and segment the ROI through the application of mathematical models and statistical methods. These algorithms substantially improve efficiency and consistency of segmentation but encounter significant limitations when applied to complex brain regions or in the presence of substantial pathological changes (Puzio et al., 2025; Yu et al., 2025). (4) Hybrid method: This integrative approach synergistically combines the strengths of manual labeling and automatic segmentation algorithms. For example, initial manual contours can inform and constrain the automatic segmentation process, with subsequent refinements and iterative optimizations based on the algorithm’s results (Parmar et al., 2014).

The selection of an appropriate method should be determined by the research objectives, the intrinsic characteristics of the data, and the available research resources. Segmentation accuracy and methodological reproducibility constitute the fundamental criteria for evaluating the performance of these segmentation approaches.

### 2.3 Extraction of features and construction of prediction model

Features can be broadly categorized into qualitative and quantitative categories (Cheung et al., 2022). Qualitative features include specific location, size, morphology, etc. Quantitative features can be systematically subdivided into the following four types: (1) Morphological Features: These features elucidate the three-dimensional shape and structural features of the lesion (Kumar et al., 2012); (2) First-Order Features (Histogram Features): These features represent the distribution of the lesion across different gray levels, providing quantitative metrics regarding of the lesion’s intensity characteristics (Zwanenburg et al., 2020); (3) Second-Order Features: These quantify the heterogeneity of the lesion by examining the spatial relationships between voxels or pixels, thereby capturing complex textural patterns and architectural information (Zwanenburg et al., 2020); (4) Higher-Order features: These comprise advanced mathematical transformations including Wavelet transforms, Laplace filters, and Gabor filters, etc. which facilitate multi-scale analysis of the lesion at a more sophisticated level, elucidating subtle patterns and structures (Kumar et al., 2012; Zhang et al., 2022; Zwanenburg et al., 2020). When performing radiomics analysis, the focus is on identifying various image attributes including shape, intensity, texture, gradient, and wavelet. Furthermore, the incorporation of non-image data encompassing clinical information and biogenetic data should be systematically evaluated to optimize model performance and clinical relevance.

The construction of the prediction model often employs algorithmic models based on machine learning, such as convolutional neural networks, support vector machines (SVM), and random forests, representing a critical component in the radiomics workflow (Lambin et al., 2017; Rathore et al., 2017). Sensitivity, specificity, accuracy, and the receiver operating characteristic (ROC) curve analysis constitute the standard metrics for rigorous evaluation of the model (Bera et al., 2022; Lambin et al., 2017). Ultimately, these metrics serve as the cornerstone for validating the reliability and performance of our predictive models in clinical applications.

## 3 Advances in radiomics of Alzheimer’s disease

Alzheimer’s disease (AD) is the most common neurodegenerative disorder, which has a serious impact not only on the patient’s daily life but the psychological and economic status of the patient’s family (Alzheimer’s Association, 2016). Mild cognitive impairment (MCI) is characterized as the transitional stage from normal cognition to AD (Dubois et al., 2016) with approximately 10–20% of MCI patients progressing to dementia annually (Langa, Levine, 2014). Studies have demonstrated that therapeutic intervention during the MCI stage may delay the onset of irreversible dementia (Mueller et al., 2005). Therefore, there exists an urgent need for reliable biomarkers to facilitate early screening and diagnosis of AD. However, volumetric alterations of the brain regions remain subtle and challenging to detect during the early stage of AD. Therefore, the identification of alternative biomarkers for the early prediction of MCI-to-AD conversion is imperative.

In recent years, neuroimaging measures (volume, thickness, surface morphometry) derived from structural magnetic resonance imaging (MRI), functional MRI, and positron emission tomography (PET) have been proposed as reliable biomarkers of AD. Radiomics analysis incorporating these parameters has been demonstrated significant potential for the automated diagnosis of MCI progression to AD. Cheung et al. constructed a model using brain region volumes and radiomics features separately (Cheung et al., 2022). A total of 107 whole-brain radiomics features were extracted using voxel-based morphometry (VBM) and the DARTEL algorithm from structural MRI data. Their findings indicated that features of the whole-brain derived from T1-weighted MR images achieved excellent performance in differentiating between AD and MCI. Besides, the hippocampus are also often used as the ROI in studies of cognitive disorders. Sorensen and colleagues (Sørensen et al., 2017) used hippocampal texture, hippocampal shape and cortical thickness to differentiate between MCI and AD based on T1-weighted structural MRI scans. A total of 215 radiomics texture features were extracted from the hippocampus, with sequential forward feature selection subsequently applied for feature selection and model construction. The analysis revealed hippocampal texture as the most significant discriminative feature, suggesting its diagnostic utility operates independently of hippocampal volume. Ranjbar et al. (2019) received similar conclusions. In their analysis of 173 subjects comprising AD, MCI, and healthy control groups, hippocampus texture features acquired the favorable performance in distinguishing AD from controls with an area under curve (AUC) of 0.89. While the hippocampus have remained a focal region in AD radiomics research, Chen et al. (2025) explored the potential of cerebellar-derived radiomics for predicting AD progression over a 6-year follow-up with integrated machine learning models. Notably, the cerebellar models outperformed hippocampal models in distinguishing MCI and in predicting transitions from normal cognition to MCI. Key predictors included textural features in the right III and left I and II lobules, as well as network properties in Vermis I and II, which were significantly associated with cognitive decline in AD. In conclusion, the whole-brain, hippocampus and cerebellum are regarded as ROI in different researches, more studies are required to ensure the optimal choice.

In terms of the model construction, several machine learning methods have been used for distinguishing MCI conversion to AD. Luk et al. (2018) utilized SVM in distinguishing MCI conversion to AD and achieved high accuracy. Feng et al. (2018) constructed a logistic regression model using t-test, correlation analysis, and LASSO screening, achieving an AUC of 0.720. Tang’s team (Tang et al., 2021) followed 162 patients with MCI, and after 5 years of follow-up, 68 patients transitioned to AD. They built a radiomics model using LASSO Cox regression analysis. The C-index was 0.950 (0.929–0.971). Lin et al. (2025) developed and validated a clinical-radiomics integrated nomogram model. The radiomics features from T1-weighted MRI images combined with clinical factors identified through univariate and multivariate Cox regression, were used to construct clinical, radiomics, and integrated models. The integrated model demonstrated superior predictive performance, achieving a C-index of 0.903 (95% CI: 0.870–0.936) in the training cohort and 0.813 (95% CI: 0.734–0.892) in the validation cohort. In a different imaging modality, Jiang et al. (2022) proposed a workflow for radiomics predictive modeling analysis of AD using fluorodeoxyglucose positron emission tomography (FDG PET) images. A total of 34,400 quantitative features were extracted from 80 cortical regions in each subject. The machine learning methods they used were t-test and LASSO. Three models were constructed: a clinical model, a standardized uptake value ratio (SUVR) Cox model, and a radiomics-based predictive model (RPM model). This study confirmed that metabolic profiles of AD pathologically susceptible regions are more effective in predicting progression to AD, and that metabolic abnormalities in these regions are better characterized by high-dimensional radiomics features (Table 1 presents a summary of the above studies).

**Table 1:** Summary of radiomics studies predicting progression of MCI to AD.

First authors	Number of subjects	Image acquisition method	Pre-processing: yes/no	Region of interest (ROI) and segmentation	Feature selection method	Model construction method	Model evaluation metric	Conclusions
Cheung et al. (2022)	512(AD = 97, MCI = 293, NC = 192) from the ADNI database	T1- weighted MRI images	Yes	Whole-brain	Not specified	Random Forest	Accuracy, Sensitivity, Specificity, Area under ROC curve.	The radiomics features from T1-weighted MR images achieved excellence performance in differentiating AD, MCI and NC.
Sørensen et al. (2017)	504 (AD = 101, MCI = 234, NC = 169) from ADNI database, 145 (AD = 28, MCI = 29, NC = 88) from AIBL database and 30 (AD = 9, MCI = 9, NC = 12) from CAD Dementia database	T1- weighted MRI images	Yes	Hippocampus (manually)	Sequential forward feature selection, Pearson correlation	Support vector machine (SVM) with a radial Gaussian kernel	Accuracy, Sensitivity, Area under ROC curve.	This study highlighted the importance of hippocampal texture as a feature in the algorithm in order to discriminate NC, MCI, and AD simultaneously based on a single structural MRI scan.
Ranjbar et al. (2019)	173 (AD = 41, MCI = 70, NC = 62) from ADNI database	T1- weighted MRI images	Yes	Hippocampus	Student t test, Pearson chi-square test	Diagonal quadratic discriminant analysis (DQDA)	Area under ROC curve.	The study supported the use of brain MR radiomics features to identify early cognitive impairment and the clinical utility of MR texture features as biomarkers of Alzheimer’s disease.
Chen et al. (2025)	1,319 (AD = 215, MCI = 530, NC = 574) from ADNI database; 308 (AD = 102, MCI = 104, NC = 102) from in-house database	T1- weighted MRI images	Yes	Cerebellar and Hippocampus	Minimum Redundancy Maximum Relevance (mRMR)	Support vector Machine (SVM), Linear discriminant analysis. Autoencoder, Random forest, Logistic regression, Logistic regression via LASSO, AdaBoost, decision tree, Gaussian process, and Naive Bayes.	Area under ROC curve.	Cerebellum-derived radiomic-network modeling shows promise as a tool for early identification and prediction of disease progression during the preclinical stage of AD.
Luk et al. (2018)	790 (AD = 183, MCI = 382, NC = 225) from ADNI database	T1- weighted MRI images	Yes	Whole-brain	F-test	Binary logistic regression model	Area under ROC curve.	Whole-brain 3D texture analysis had potential to predict progression from MCI to AD.
Feng et al. (2018)	122 (AD = 78, NC = 44) from a local cohort	3D magnetization prepared rapid gradient echo images (3D MP-RAGE images)	No	Corpus callosum (manually)	T-test and the rank test, Correlation analysis, Least Absolute Shrinkage and Selection Operator (LASSO)	Logistic regression model	Area under ROC curve, Sensitivity, Specificity, Accuracy, Precision, and Positive and negative predictive values.	Corpus callosum texture features based radiomics model was valuable for the diagnosis of AD.
Tang et al. (2021)	162 MCI from ADNI database	T1- weighted MRI images and T2-weighted fluid-attenuation inversion recovery MRI images	Yes	Bilateral hippocampus and 12 subcortical nuclei (manually)	Spearman rank correlation, Univariate cox analysis, LASSO and 10-fold cross validation	LASSO cox regression	Area under ROC curve, C-index.	The prediction of individual time to progression from MCI to AD could be accurately conducted using the radiomics-clinical-laboratory model and multi predictor nomogram.
Lin et al. (2025)	244 (preclinical AD = 51, NC = 193) from ADNI database	T1- weighted MRI images	Yes	Gray matter, White matter, and Cerebrospinal fluid (automatically segmented)	LASSO regression, Spearman rank correlation	Cox proportional hazards model.	C-index.	The study constructed a clinical-radimoics integrated model to predict the progression of preclinical AD and stratify the risk of disease progression in preclinical AD.
Jiang et al. (2022)	Cohort I (sMCI = 18, pMCI = 168, NC = 94) from ADNI database; Cohort II (sMCI = 81, pMCI = 3) from the ADNI-Go database; Cohort III (NC = 138, SCD = 76, MCI = 41, AD = 60) from local hospitals.	FDG PET images	Yes	80 cortical regions from the automated anatomical labelling (AAL) atlas	T-test, LASSO	Proportional hazards model	C-index	The preliminary results demonstrated that the developed radiomics-based predictive model had the potential to monitor progression in high-risk populations with AD.

AD, Alzheimer’s disease; MCI, Mild Cognitive Impairment; NC, Normal Control; ADNI, Alzheimer’s Disease Neuroimaging Initiative; AIBL, Australian Imaging Biomarkers and Lifestyle flagship study of ageing; CAD Dementia, Computer-Aided Diagnosis of Dementia; AUC, area under the ROC curve; SVM, Support Vector Machine; DQDA, The diagonal quadratic discriminant analysis; MP-RAGE, magnetization prepared rapid gradient echo; LASSO, Least Absolute Shrinkage and Selection Operator; sMCI, stable MCI (refer to patients fulfill MCI diagnostic criteria but demonstrate no significant progression during 3 years follow-up); pMCI, progressive MCI (refer to patients fulfill MCI diagnostic criteria and transfer to AD during 3 years follow-up); ADNI-Go, Alzheimer’s Disease Neuroimaging Initiative grand opportunities; SCD, subjective cognitive decline; FDG PET, fluorodeoxyglucose positron emission tomography; AAL, Automated Anatomical Labeling; PET, positron emission tomography.

The application of radiomics in AD can provides insights into the mechanisms and pathological processes of AD, which facilitates early diagnosis and prediction. Recent studies highlight the potential of radiomics and machine learning in predicting AD progression from MCI using neuroimaging biomarkers. Key brain regions including the hippocampus, cerebellum, and precuneus provide discriminative features. While hippocampal textural features outperforms volumetry, emerging biomarkers in the cerebellum and precuneus challenge traditional AD paradigms. However, current limitations include small sample sizes, unclear biological correlates of radiomic features, and limited clinical generalizability. Future research should focus on longitudinal validation, multimodal integration, and explainable AI to enhance translational utility.

## 4 Advances in radiomics for predicting cognitive impairment in Parkinson’s disease

Parkinson’s disease (PD) is a progressive neurodegenerative disorder that primarily affects dopamine-producing neurons in the specific region of the brain called the substantia nigra (SN). Diagnosis is typically based on clinical history and motor symptoms (Chu et al., 2024). However, the early-stages of PD often present diagnostic challenges due to heterogeneous clinical presentations and overlapping symptoms with other neurological disorders. These challenges are particularly pronounced when evaluating cognitive impairments associated with PD, which are often subtle and difficult to differentiate. To address these complexities, radiomics has emerged as a promising approach for early diagnosis through the extraction and analysis of high-throughput quantitative imaging features. This advanced approach not only facilitates in the detection of PD but also holds potential for identifying and characterizing cognitive impairments, thereby enhancing both diagnostic accuracy and disease management.

Cognitive impairment is one of the most common non-motor symptoms of PD (Aarsland et al., 2017). PD dementia (PDD) occurs in more than 80% of PD patients with disease duration exceeding 20 years (Hely et al., 2008). PDD primarily affects cognitive domains such as attention, executive function, and working memory. PD-MCI is prevalent in 19–42% of PD patients (Aarsland et al., 2009; Yarnall et al., 2014) and it is usually considered a risk factor for PDD (Pedersen et al., 2013). However, substantial heterogeneity exists in the cognitive trajectories of PD-MCI, some patients progress to PDD, while others remain stable or even return to normal cognition (Pedersen et al., 2013). Therefore, the risk stratification of PD-MCI convention to PDD is essential, as high-risk patients may require more aggressive treatment and could potentially benefit from disease-modifying therapies.

Conventional structural MRI sequences were used in the evaluation of PD related cognitive impairment. Shin et al. (2021) developed a machine-learning-based predictive model to assess the risk of progression to dementia in patients with PD-MCI using cortical thickness measurements based on T1-weighted MR images. Train random forest and SVM models were used to construct the model. The study demonstrated that radiomics features derived from the cortical thickness on conventional structural MRI sequences could predict the progression from MCI to dementia in individual PD patients. More recently, advanced multiparametric radiomics models have further improved predictive accuracy and interpretability. Park et al. (2023) analyzed T1, T2, and T2-FLAIR sequences from basal ganglia MRI and extracted more than 1,200 radiomic features. Their multiparametric radiomics model achieved an AUC of 0.928, identifying key radiomic features associated with dementia progression.

Increased iron deposition in the SN system is one of the most common features of PD. Evidence suggests that cognitive impairment is often secondary to abnormal brain tissue iron deposition and this mechanism may also contribute to the pathology of PDD (Uchida et al., 2019). Therefore, imaging markers that quantify iron deposition have received increasing attention in recent years (An et al., 2018). Magnetic susceptibility value (MSV) derived from quantitative susceptibility mapping (QSM) can quantify iron deposition while radiomics can analyze the distribution of iron deposition. Kang et al. (2022) explored whether MSV and radiomics features could serve as imaging markers for the objective assessment of CI in PD patients. The study used SN, head of the caudate nucleus (HCN), and putamen as the ROI. Multivariate logistic regression and SVM models were developed. The study revealed that characteristics including mean absolute difference, variance, and gray-scale variance of the HCN in PD patients were negatively correlated with the Montreal Cognitive Assessment (MoCA) scores. Similar to the previous study, Zhao et al. (2022) used QSM to evaluate iron deposition and microstructural changes in 60 patients with PD (16 with MCI). They performed voxel-based and radiomic analysis of subcortical nuclei (substantia nigra, basal ganglia) to extract texture and susceptibility-related features. A SVM classifier was able to distinguish cognitively normal PD patients from PD-MCI ones with 83% accuracy, suggesting that QSM-based radiomics represents a sensitive biomarker for early cognitive impairment in PD.

Diffusion tensor imaging (DTI) scan was also used in the prediction of PD-MCI and PDD. In a multicenter study conducted by Jian et al. (2024), the investigators developed an MRI-based radiomics model based on T1-weighted and DTI. They extracted 3,396 radiomic features focusing on gray and white matter regions. The feature selection method was LASSO regression and the model construction method was random forest model. The model achieved an AUC of 0.86. Zeng et al. (2025) investigated hippocampal functional imaging with resting-state fMRI in 89 PD patients (55 with cognitive impairment). Their research results suggest the logistic regression model achieved 88.9% accuracy, emphasizing hippocampal functional connectivity detected by resting-state fMRI as a key predictor of cognitive decline of PD patients.

Beyond structural changes, cognitive impairment in PD has also been linked to disruptions in specific neural circuits. Previous research suggests that CI is associated with dysfunction in frontal-striatal circuits, frontal-basal ganglia circuits, and frontal-thalamic circuits in patients with PD (Apostolova et al., 2010; Kim et al., 2012). Supporting this, functional and molecular imaging studies have further explored radiomic correlates of CI. For instance, Rahmim et al. used single-photon emission computed tomography (SPECT) radiomic features to investigate the correlation between striatal dopamine transporter and PD-MCI (Rahmim et al., 2016). This study suggested that SPECT could also be used as the tool for objectively assessing CI in PD patients (Table 2 presents a summary of the above studies).

**Table 2:** Summary of Radiomics Studies Predicting Cognitive Impairment in PD.

First authors	Number of subjects	Image acquisition method	Pre-processing: yes/no	Region of interest (ROI) and segmentation	Feature selection method	Model construction method	Model evaluation metric	Conclusions
Kang et al. (2022)	149 (PD = 104, NC = 45) from a local cohort	T1-weighted MRI images, T2-weighted MRI images, and T2 fluid-attenuated inversion recovery MRI images	Yes	Substantia nigra, Head of caudate nucleus, and Putamen	LASSO regression	MLR and SVM	Area under ROC curve	Radiomics features and magnetic susceptibility value of the nigrostriatal system from quantitative susceptibility mapping could have a crucial role in diagnosing PD and assessing CI.
Zhao et al. (2022)	60 (PD-MCI = 16, PD-NC = 16, NC = 28) from a local cohort	T1-weighted MRI images, T2-weighted MRI images, and multi-gradient echo-based QSM sequences.	Yes	Substantia nigra, Hippocampus	Not specified	Not specified	Area under ROC curve, sensitivity, specificity	Magnetic resonance quantitative susceptibility mapping combined with voxel-wise and radiomic analysis can assess mild cognitive impairment in Parkinson’s disease.
Jian et al. (2024)	183 (PD-MCI = 50, PD-NC = 133)from PPMI database;49 (PD-MCI = 18, PD-NC = 21)	T1-weighted MRI images, Diffusion tensor imaging sequence	Yes	Gray matter, White matter, and Cerebrospinal fluid (automatically segmented)	Minimum redundance maximum relevance (mRMR), and LASSO regression	Logistic regression model	Area under ROC curve	MRI radiomics combined with clinical features can predict cognitive decline in Parkinson’s disease.
Shin et al. (2021)	117 (PDD = 42, PD-MCI = 75) from a local cohort	T1-weighted MRI images	Yes	Cortical thickness	LASSO regression	Random forest and SVM	Area under ROC curve	Cortical thickness from MRI helped predict conversion from mild cognitive impairment to dementia in Parkinson’s disease at an individual level, with improved performance when integrated with clinical variables.
Park et al. (2023)	262 (Among them, PDD = 75) from a local cohort	T1-weighted MRI images, T2-weighted MRI images, and T2 fluid-attenuated inversion recovery MRI images	Yes	Frontal/executive function domain, Caudate	Ten-fold cross validation	Multiparametric radiomics model	Area under ROC curve, accuracy, sensitivity, specificity	An interpretable multiparametric radiomics model of basal ganglia can predict dementia conversion in Parkinson’s disease.
Zeng et al. (2025)	89 (PD-CI = 55, PD-NC = 34) from a local cohort	T1-weighted MRI images, T2-weighted MRI images, T2 fluid-attenuated inversion recovery MRI images, High-resolution 3D T1-weighted structural images, and the gradient-recalled echo echo-planar imaging sequence	Yes	Bilateral hippocampi	Spearman rank correlation, LASSO regression	Logistic regression models	Area under ROC curve, sensitivity, and specificity	Hippocampal functional imaging-derived radiomics features can diagnose cognitively impaired patients with Parkinson’s disease.
Rahmim et al. (2016)	122 (PD = 85, NC = 56) from PPMI database	Dopamine transporter (DAT) SPECT images; High-resolution MRI images	Yes	Boundaries of the caudate and putamen (both left and right)	Pearson correlation, Multivariate stepwise linear regression analysis	Multivariate stepwise linear regression analysis	Pearson correlation coefficient	The results demonstrated the ability to capture valuable information using advanced texture metrics from striatal DAT SPECT, enabling significant correlations of striatal DAT binding with cognitive outcomes.

PD, Parkinson’s disease; PUT, putamen; LASSO, The least absolute shrinkage and selection operator; QSM, Quantitative susceptibility mapping; LR, Multivariate logistic regression; SVM, support vector machine; PDD, Parkinson disease dementia; PD-CI, Parkinson disease with cognitive impairment; PD-MCI, Parkinson disease with mild cognitive impairment; PD-NC, Parkinson disease with normal cognition; PPMI, Parkinson’s progression markers initiative.

In conclusion, radiomics shows promise in predicting cognitive impairment in PD, capturing subtle brain changes and enhancing diagnostic precision. Integration with clinical data improves predictive accuracy, but challenges persist. Model heterogeneity and lack of interpretability hinder clinical adoption. Future research should address these issues to develop robust, interpretable, and generalizable radiomics models for PD cognitive impairment prediction.

## 5 Advances in radiomics for predicting cognitive impairment associated with cerebrovascular disease

As life expectancy continues to increase, age-related diseases have become one of the most significant health challenges worldwide. Cerebral small vessel disease (CSVD) is a common age-related condition that affects 80% of the elderly population are suffering (Zhang et al., 2023). CSVD is recognized as one of the major causes of stroke and vascular cognitive dysfunction, significantly impacting patients’ quality of life (Cannistraro et al., 2019). The pathogenesis of CSVD-related cognitive dysfunction remains incompletely understood, however, early intervention can delay or even reverse its progression (Ngandu et al., 2015).

With advances in neuroimaging techniques, multiparametric MRI plays a crucial in the early diagnosis of CSVD-related cognitive dysfunction. These techniques can non-invasively assess neuroimaging indices of brain structure, function, perfusion, and metabolism. Comprehensive analysis of multiparametric MRI data using machine learning can facilitate accurate diagnosis and prognostic assessment of CSVD at the individual level. Türe et al. (2000) reported that the majority of pathological changes associated with CSVD occur in the basal ganglia and corona radiata regions supplied by the lenticulostriate artery (LSA). These regions affect basic motor, sensory, and visual functions. Additionally, a recent study (Decavel et al., 2012) reported that cognitive deficits occur in approximately 30–80% of LSA infarction cases, necessitating analysis of the potential relationship between LSA and cognitive deficits. Zhou et al. (2024) collected data from 102 CSVD patients with cognitive dysfunction and developed a model that combined LSA radiomics with clinical features using high-resolution magnetic resonance black blood sequences, which aimed to analyze the potential relationship between corresponding LSA imaging markers and cognitive dysfunction. The study selected four clinical features (left hemisphere stems, left hemisphere branches, bilateral stems, total vessel count) and six wavelet transform features using LASSO regression analysis. Three models were constructed based on clinical features, radiomics features, and combined clinical-radiomics features, respectively. The study demonstrated that the combined model achieved the best predictive performance, with the radiomics model also outperformed the clinical model.

Post-stroke cognitive impairment (PSCI) is a major component of stroke sequelae (Pantoni, Salvadori, 2021), which includes ischemic PSCI (iPSCI) and hemorrhagic PSCI (hPSCI) each with distinct pathogenesis. iPSCI stems from ischemic cerebral infarction disrupting the cortico-subcortical network, particularly in the frontal-executive circuit. In contrast, hPSCI results from direct tissue destruction via hematoma mass effect with secondary iron-mediated neurotoxicity, neuroinflammation and subarachnoid hemorrhage (SAH)-associated delayed cerebral ischemia. Despite their distinct mechanisms, both iPSCI and hPSCI represent critical forms of post-stroke cognitive dysfunction requiring early intervention to prevent irreversible decline. Studies have shown that the interval between the onset of stroke and PSCI development represents a therapeutic window for early intervention to preserve cognitive function (Brainin et al., 2015). Therefore, identifying reliable predictors of PSCI is essential for improving long-term stroke outcomes. For iPSCI, radiomics analysis of white matter integrity can capture subtle microstructural changes prior to cognitive decline (Habes et al., 2016). In hPSCI, perihematoma lesion heterogeneity quantified through CT/MRI radiomics (Haider et al., 2023) may predict the risk of iron-mediated neurotoxicity. Such imaging biomarkers may enable targeted interventions during this vulnerable stage, ultimately improving long-term cognitive outcomes.

The texture features derived from routine clinical MR images can represent robust early predictors of PSCI. Betrouni et al. (2022) analyzed texture features of the hippocampus and entorhinal cortex to predict cognitive status 6 months post-stroke using a random forest model based on T1-weighted images. The model achieved an accuracy of 0.90 ± 0.05, sensitivity was 0.92 ± 0.04, specificity was 0.93 ± 0.02, and AUC for the subject’s working characteristics was 0.90 ± 0.03 in the study.

Several studies have leveraged radiomics and functional connectivity (FC) analyses to uncover early predictive markers of PSCI. Dragoș et al. (2025) enrolled 144 patients to investigate the predictive role of FC and MRI radiomic markers in evaluating PSCI 1 year after acute ischemic stroke (AIS). The study employed MRI and electroencephalogram (EEG) processing techniques to assess brain and cognitive reserve. By integrating these measures with predictive models based on quantitative EEG (QEEG), MRI radiomics, and clinical data, the study sought to identify early predictive factors for PSCI using Cox proportional hazards models. The integration of FC and radiomics biomarkers offers potential for enhanced predictive accuracy, possibly facilitating more effective therapeutic approaches for PSCI. Besides, Weaver et al. (2021) conducted a large multicohort study including 2,950 patients were enrolled, with MRI imaging data and cognitive assessments were obtained up to 15 months after AIS onset. The study selected the infarcts in the left frontotemporal lobe, right parietal lobe, and left thalamus as the ROI and got the conclusion that the occurrence of PSCI could be predicted using a voxel-based lesion-symptom mapping (VLSM) model. This study underscores the importance of lesion location in predicting PSCI, complementing the findings of Dragoș et al. by emphasizing the structural correlates of PSCI. Miao et al. (2021) combined resting-state functional magnetic resonance imaging (rs-fMRI) with radiomics features to investigate FC of low-degree rich club organization and the caudate nucleus. The results revealed that changes in functional connectivity of low-degree rich club organization and the caudate nucleus were correlated with 3D shape features and first-order statistics of stroke lesions. These radiomic features may indicate disruption of FC and low-degree rich club organization, serving as potential imaging markers of PSCI and providing new insights into its neural mechanisms (Table 3 presents a summary of the above studies).

**Table 3:** Summary of Radiomics Studies Predicting Cognitive Impairment in Cerebrovascular disease.

First authors	Number of subjects	Image acquisition method	Pre-processing: yes/no	Region of interest (ROI) and segmentation	Feature selection method	Model construction method	Model evaluation metric	Conclusions
Zhou et al. (2024)	120 (MCI = 58, MSCI = 44) from a local cohort	T1-weighted MRI images, High-resolution magnetic resonance black blood sequences MRI images	Yes	Entire lenticulostriate artery (LSA) vasculature (manual)	The max-relevance and min-redundancy, Five-fold cross-validation and LASSO	Multivariate logistic regression	Area under ROC curve	The model that combines clinical and radiomics features of LSA could predict MCI.
Dragoș et al. (2025)	144 stroke patients from a local cohort	T1-weighted MRI images, T2 fluid-attenuated inversion recovery MRI images, diffusion-weighted MRI images	Yes	The AIS lesion	Not specified	Cox proportional hazards model,	Area under ROC curve, Accuracy	A predictive model is constructed based on quantitative electroencephalogram, MRI radiomics and clinical data, which can identify patients with acute ischemic stroke who are prone to develop PSCI at an early stage.
Weaver et al. (2021)	2,950 stroke patients from 12 local cohorts	T1-weighted MRI images, T2 fluid-attenuated inversion recovery MRI images, diffusion-weighted MRI images	Yes	The left frontotemporal lobes, left thalamus, and right parietal lobe	Leave-one-cohort-out cross-validation	Multivariate logistic regression model	Not specified	The occurrence of PSCI could be predicted by a voxel-based lesion-symptom mapping (VLSM) model.
Betrouni et al. (2022)	327 stroke patients from the STROKOG members	T1-weighted MRI images	Yes	The hippocampus and the entorhinal cortex	Not specified	Random forest	Accuracy, Sensitivity, Specificity	These results suggested that texture features obtained from routine clinical MR images were robust early predictors of poststroke cognitive impairment.
Miao et al. (2021)	58 (HC = 21, hPSCI = 16, iPSCI = 21) from a local cohort	T1-weighted MRI images, Resting-state MRI images	Yes	Caudate Nucleus	Pearson’s correlation	Unmodeled	Intraclass correlation coefficient	The radiomic features of stroke lesions may suggest FCs damage and low abundant club organization, which are potential imaging markers of PSCI and provide new insights into the neural mechanisms of PSCI.

MCI, mild cognitive impairment; MSCI, moderate or severe cognitive impairment (MoCA score≤17); ROC, Receiver Operating Characteristic Curve; LSA, lenticulostriate artery; AIS, acute ischemic stroke; PSCI, post stroke cognitive impairment; VLSM, voxel-based lesion-symptom mapping; STROKOG, Stroke and Cognition Consortium from centers; hPSCI, hemorrhagic stroke post-stroke cognitive impairment; iPSCI, infarct stroke post-stroke cognitive impairment.

These studies demonstrate the potential of radiomics as a robust tool for predicting cognitive outcomes following stroke. By leveraging multiparametric MRI and machine learning, radiomics models can identify early predictive markers of cognitive dysfunction. The integration of radiomics with functional connectivity and clinical data enhances predictive accuracy, providing insights into the neural mechanisms underlying cognitive decline. However, challenges remain, including the need for larger, diverse cohorts to validate the findings and the complexity of interpreting radiomics features in the clinical background.

## 6 Discussion

The above studies highlight the potential of radiomics to extract high-dimensional quantitative features from medical images. The recent studies integrate radiomics with clinical and neuropsychological data, thereby enhance the predictive accuracy of the models. This multidimensional approach allows for a more comprehensive assessment of cognitive decline, capturing both imaging-derived and clinical risk factors. The application of machine learning techniques in radiomics analysis enables the development of sophisticated predictive models. These models can identify complex patterns and relationships within the data, facilitating early diagnosis and risk stratification of cognitive impairment.

The existing studies also exhibit several limitations. First, significant heterogeneity exists in the imaging protocols, patient populations, and cognitive assessment tools employed across studies. Standardization of imaging protocols and cognitive assessments is essential to enhance the comparability of radiomics-based studies. Second, many studies are limited by small sample sizes, which may diminish both statistical power and generalizability of the findings. Larger, prospective studies are needed to validate the predictive value of radiomics biomarkers. Third, while little studies employ longitudinal follow-up, many existing researches are cross-sectional, thereby restrict the ability to understand the dynamic changes of cognitive decline. Fourth, despite the high predictive accuracy of radiomics models, their interpretability remains challenging. Future researches should focus on developing interpretable radiomics models that can be easily integrated into clinical practice.

## 7 Conclusion

The strength of radiomics lies in its capacity to systematically and efficiently extract complex information from medical images, thereby providing novel perspectives and valuable tools for the realms of medical diagnosis and disease management. Radiomics-derived imaging biomarkers, particularly those targeting cognitive dysfunction, represent important clinical applications. These biomarkers enable physicians to efficiently differentiate patients with cognitive disorders across a spectrum of neurological conditions, facilitating timely and appropriate interventions. Nevertheless, the field of radiomics continues to face several challenges, including index standardization and normalization, limited sample sizes, and data consistency of data across multicenter studies. Therefore, further research and technological advancement are required to enhance the effectiveness and reliability of radiomics in cognitive impairment research.
